# Translation and validation of the German version of the Bournemouth Questionnaire for Neck Pain

**DOI:** 10.1186/2045-709X-20-2

**Published:** 2012-01-25

**Authors:** Marina Soklic, Cynthia Peterson, B Kim Humphreys

**Affiliations:** 1 University of Zürich Chiropractic Medicine Programme, Orthopaedic University Hospital Balgrist. Forchstrasse 340, 8008 Zürich Switzerland; 2 University of Zürich and Orthopaedic University Hospital Balgrist. Forchstrasse 340, 8008 Zürich Switzerland

**Keywords:** Bournemouth Questionnaire, Outcome Assessment, Neck Pain, Chiropractic, Validity of Results

## Abstract

**Background:**

Clinical outcome measures are important tools to monitor patient improvement during treatment as well as to document changes for research purposes. The short-form Bournemouth questionnaire for neck pain patients (BQN) was developed from the biopsychosocial model and measures pain, disability, cognitive and affective domains. It has been shown to be a valid and reliable outcome measure in English, French and Dutch and more sensitive to change compared to other questionnaires. The purpose of this study was to translate and validate a German version of the Bournemouth questionnaire for neck pain patients.

**Methods:**

German translation and back translation into English of the BQN was done independently by four persons and overseen by an expert committee. Face validity of the German BQN was tested on 30 neck pain patients in a single chiropractic practice. Test-retest reliability was evaluated on 31 medical students and chiropractors before and after a lecture. The German BQN was then assessed on 102 first time neck pain patients at two chiropractic practices for internal consistency, external construct validity, external longitudinal construct validity and sensitivity to change compared to the German versions of the Neck Disability Index (NDI) and the Neck Pain and Disability Scale (NPAD).

**Results:**

Face validity testing lead to minor changes to the German BQN. The Intraclass Correlation Coefficient for the test-retest reliability was 0.99. The internal consistency was strong for all 7 items of the BQN with Cronbach α's of .79 and .80 for the pre and post-treatment total scores. External construct validity and external longitudinal construct validity using Pearson's correlation coefficient showed statistically significant correlations for all 7 scales of the BQN with the other questionnaires. The German BQN showed greater responsiveness compared to the other questionnaires for all scales.

**Conclusions:**

The German BQN is a valid and reliable outcome measure that has been successfully translated and culturally adapted. It is shorter, easier to use, and more responsive to change than the NDI and NPAD.

## Background

Musculoskeletal problems are extremely common in our population, especially neck pain and its associated disability [1]. The therapy for neck pain includes relieving of pain, stiffness and disability through treatments which may include exercise, traction, acupuncture, mobilization and manipulation [2,3]. To determine whether or not specific treatments are effective for the various causes of neck pain, appropriate patient outcomes must be recorded.

Clinical outcome measures such as self-report questionnaires are useful in monitoring patient improvement during treatment. The vast majority of disease-specific instruments have been developed in English- speaking countries [4]. The most commonly used neck pain specific questionnaires are the Neck Disability Index (NDI) [5], the Northwick Park Neck Pain Questionnaire [6], the Copenhagen Neck Functional Disability Scale [7], the Neck Pain and Disability Scale (NPAD) [8], and the Bournemouth Questionnaire for Neck Pain (BQN) [9]. The NDI is the most commonly used instrument in neck pain research [9].

Neck pain, similar to low back pain is a multidimensional experience, best described by the biopsychosocial model that includes pain, disability, cognitive and affective domains [9]. However, many of the current neck pain questionnaires such as the NDI measure only pain and disability. The BQN was developed from the biopsychosocial model and includes questions on psychosocial issues as well as pain and disability. The BQN is a short-form, multidimensional instrument originally created in English, that has been shown to be valid, reliable and responsive for use in the clinical and research settings [9].

In order to use the BQN in a German speaking patient population it is not enough to just translate the items well linguistically, because that does not guarantee similar measurement properties [10]. The questionnaire also has to be adapted cross-culturally, which means employing a process that looks at both language and cultural issues relevant to the German speaking population in which the questionnaire will be used [4].

As the BQN is only available in English, French and Dutch [9,11,12], the purpose of this study was to translate and validate a German version.

## Methods

Ethics approval for this study was obtained by the Canton of Zürich ethics review board (KEK-ZH-Nr.2010-0252/5).

### Translation

**Figure 1:**
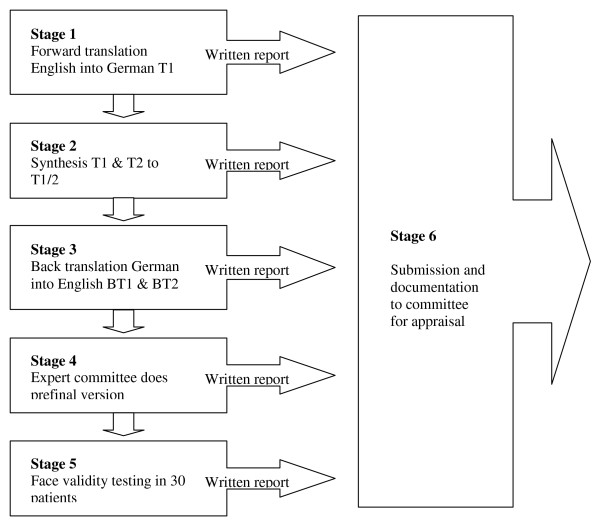
**Translation and Cross-Culture Adaptation Sequence**.

The translation and cross cultural adaptation were based on the guidelines of Beaton, Bombardier et al. [4]. The entire process is made up of 6 steps (Figure 1).

#### Stage 1

Two translators (T1 and T2) translated independently the questionnaire from English into German. Both T1 and T2 had German as their mother language but were also fluent in English. One of the translators (T1) is a chiropractor in Switzerland (clinician) and the second (T2) is a Swiss librarian (linguist). They both provided a written report.

#### Stage 2

The two independent translated versions TV1 and TV2 were revised by consensus agreement to TV1-2 by the original translators, and overseen by the expert committee.

#### Stage 3

The agreed TV1-2 version was then back translated by two independent translators (BT1 and BT2). Both back translators had English as their first language but were fluent in German. BT1 and BT2 were both chiropractors from Canada working in the German speaking part of Switzerland for several years. They were blinded to the original version of the Bournemouth questionnaire. Their two versions of the back translation (BTV1 and BTV2) were submitted to the committee.

#### Stage 4

An expert committee reviewed all reports and agreed by consensus to a pre-final version of the German Bournemouth questionnaire. This team of 8 people was made up of methodologists, health professionals, language professionals, and translators. The original developer of the English version of the BQN also participated in an advisory capacity.

#### Stage 5

The pre-final form of the BQN was tested on a sample of 30 patients in a chiropractic practice in Zürich, Switzerland for face validity. The neck pain patients were asked to complete the questionnaire after having treatment. Afterwards the questionnaire was discussed with the patient item by item and they were asked to explain their understanding of the meaning of each question. The patients were also asked if they had any problems with the format, instructions, response scales or layout of the questionnaire.

#### Stage 6

A written report on the face validity of the questionnaire was sent to the expert committee. Each member of the committee also made a written report. Minor changes were agreed and the pre-final form was modified to include these changes with consensus. This version then became the final German version of the BQN for validation testing (Additional file 1).

### Test-Retest Reliability

Data for the test-retest reliability study was collected during a lecture for medical students in order to ensure that the participants did not sustain any neck trauma or undergo any treatment between completing the two questionnaires. Students with neck pain were asked to complete the German version of the BQN prior to the start of the lecture. After two hours they were asked once again to fill in the BQN, but they were not told that it would be the exact same questionnaire. To protect anonymity, the students had to write the first two letters of their mother's name and the birth year of their mother on the top of the page so that the pre-lecture and post-lecture questionnaires could be matched. In order to obtain 31 participants, this same process was repeated before and after a two hour meeting of chiropractors.

### Validation

**Figure 2:**
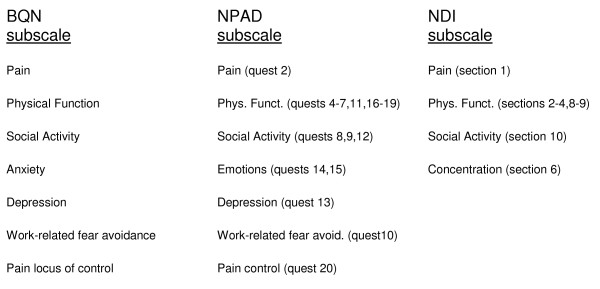
**Matching of subscales between the BQN, NPAD and NDI**.

Cross-cultural adaptation tries to ensure consistency in the content and face validity between the original and the translated versions of a questionnaire, but it does not ensure that the questionnaire has construct validity. Content validity was already specifically evaluated on the original English version of the questionnaire, and was therefore not tested in this German version. Additional testing was done to evaluate construct validity however [13,14]. This additional testing of the instrument was done in the same population where it would be used, as recommended in the literature [10]. The BQ is commonly used as an outcome measure for neck and low back pain patients being treated by chiropractors in the UK, where it was developed, as well as in other countries (9,11,13). Thus 128 neck pain patients from two different chiropractic practices were asked to fill in the new German version of the BQN, the German version of the NDI [5] and the German version of the NPAD [15] prior to the start of their chiropractic treatment-series. After finishing the treatment series or 4 weeks later, each patient had to complete the 3 questionnaires again. The questionnaires were given to them in the practice or sent by post with an addressed and stamped return envelope. Those patients who received them in the practice filled them in immediately. Those who received them by post were allowed one week to return them. If the questionnaires were not returned within 1 week, the patients were called by phone and reminded to return the completed questionnaires. If necessary, the questionnaires were resent to the patients. The NDI and NPAD were chosen as they contain similar subscales to the BQN. To compare questionnaires, each instrument was broken down into its component subscales. Figure 2 shows the matching of the various subscales on the NPAD and NDI questionnaires with the seven subscales on the Bournemouth questionnaire.

### Statistical Analysis

Test-retest reliability of the BQN was evaluated using the two way mixed Intraclass Correlation Coefficient (ICC) [10,13,16]. The internal consistency of the BQN, which measures the degree to which items that make up the total score are all measuring the same underlying attribute, was assessed using Cronbach α [10,13,16].

External construct validity shows the extent to which the BQN's scores concord with the scores of other instruments measuring the same theoretical hypotheses of the concepts under consideration [13]. This was done using the Pearson's correlation coefficient comparing the 7 scales and total score of the BQN with the NDI as well as the BQN with the NPAD for answers given at baseline (pre-treatment) and at 4 weeks after the start of treatment [14]. External longitudinal construct validity was determined with Pearson's correlation of the change scores of the various scales comparing the BQN with the other two questionnaires over the 4 week treatment period.

The sensitivity to change over time of the three questionnaires was assessed with the standardized response mean (SRM). The average change in scores for each scale was divided by the standard deviation of the score changes [13,17].

## Results

From the 128 chiropractic patients presenting with a new episode of neck pain who completed all three baseline (pre-treatment) questionnaires, 102 also provided complete 4 week post-treatment data for these same three questionnaires. Of the 102 patients included in the study, 38 were male and 64 were female with a mean age of 39.3 years (SD = 13.0). There was no significant age difference between the genders. The mean total score for the German BQN at baseline was 33.14 (SD = 15.8) or 47% of the maximum score. For the NDI the mean baseline score was 14.14 (SD = 8.0) or 28% of the maximum score and for the NPAD the mean baseline score was 35.28 (SD = 21.1) or 35% of the maximum score.

### Test-Retest Reliability of the German BQN

The ICC results from the 31 students and chiropractors participating in the test-retest reliability study indicated acceptable agreement (.91 - .99) for all scales and the total scores (table 1).

**Table 1:** Test-Retest Reliability for the German BQN. 31 patients tested.

QUESTION	ICC	95% CI	P =
1	.98	.96-.99	.0001

2	.91	.81-.96	.0001

3	.92	.83-.96	.0001

4	.93	.86-.97	.0001

5	.97	.94-.99	.0001

6	.96	.92-.98	.0001

7	.96	.91-.98	.0001

Total Score	.99	.98-.99	.0001

### Internal Consistency of the German BQN

Table 2 shows the item-corrected total correlations for the German version of the BQN, with all values well above the cut-off point of 0.2 [13], indicating that all seven scales contribute to the overall score. The Cronbach α was .79 (minimal level to establish acceptable consistency) for the total pre-treatment scores and .82 for the total post-treatment scores.

**Table 2:** Internal consistency of the German version of the BQN questionnaire

Item-Corrected Total Correlations Pearson's *r*								
Domain (Item)	1	2	3	4	5	6	7	Cronbach's alpha: Total Score
Pre-treatment	.77	.85	.81	.86	.68	.76	.63	.79

Post-treatment	.82	.86	.78	.86	.78	.81	.78	.80

### External Construct Validity and External Longitudinal Construct Validity

Table 3 contains the data for the external construct validity which compared each of the 7 scales on the BQN with similar external items from the NPAD and NDI, both at baseline and at 4 weeks after start of treatment (Figure 2). All correlations were statistically significant at p < 0.05, including the 'pain control' scale. The results for the external longitudinal construct validity of the German BQN compared to the other two questionnaires are found in table 4. These were also strongly statistically significant for all 7 scales.

**Table 3:** External construct validity of items on the German BQN.

BQ SCALE	NPAD Pre- Treatment (*r)*	NPAD Post-Treatment *(r)*	NDI Pre-Treatment *(r)*	NDI Post-Treatment *(r)*
Pain	.61	.72	.59	.65

Physical Function	.48	.56	.40	.51

Social Function	.72	.65	.67	.65

Anxiety	.55	.71	.58	.60

Depression	.52	.80		

Work-related fear avoidance	.66	.64		

Pain Control	.24	.45		

Total Score	.69	.80	.68	.76

All are significant (p < 0.05).

**Table 4:** External longitudinal construct validity of the German BQN compared with the NPAD (Neck Pain and Disability Scale) and NDI (Neck Disability Index).

BQ Neck Scale	NPAD Pearson *r *(significance)	NDI Pearson *r *(significance)
Pain	.59 (.001)	.56 (.001)

Physical Function	.36 (.001)	.37 (.001)

Social Function	.50 (.001)	.44 (.001)

Anxiety	.45 (.001)	.53 (.001)

Depression	.43 (.001)	

Work-related fear avoidance	.48 (.001)	

Pain Control	.30 (.011)	

### Standardized Response Mean (SRM)

The German version of the BQN showed greater responsiveness compared to both the NPAD and NDI for all scales (table 5).

**Table 5:** Standardized response means for the German BQN compared to the NPAD (Neck Pain and Disability Scale) and NDI (Neck Disability Index).

SCALE	BQN	NPAD	NDI
Pain	1.20	.85	1.04

Physical function	1.0	.67	.64

Social function	.86	.75	.73

Anxiety	1.1	.77	.69

Depression	.73	.32	

Work-related fear avoidance	.90	.82	

Pain control	.91	.44	

## Discussion

The Bournemouth questionnaire for neck pain (BQN) is a relatively new, short form multidimensional instrument developed from the biopsychosocial model and includes question items on pain, disability, cognitive and affective domains [9]. Currently the BQN has been translated and validated in English, French and Dutch [9,11,12]. The purpose of this study was to translate and test a German version of the BQN for use in clinical practice and research. The BQN was tested against the NDI, the most commonly used questionnaire for neck pain and the NPAD which also contains biopsychosocial questionnaire items [9]. Both the NDI and NPAD have been translated and validated in German.

The 6-step translation and cross cultural adaptation process after Beaton, Bombardier et al. [4] was used in this study and included forward and back translations, validation by an expert committee, face validity and testing in neck pain patients followed by statistical analysis. One of the analyses investigated was test-retest reliability. Although the results indicated excellent reliability (0.91-0.99), one possible source of error was the 2 hour retest time period. Terwee et al. [14] recommend that a time period of less than 1 day is too short as there is a high chance that patients can remember their previous answers. This may have been the case, however other research has confirmed a high level of test-retest reliability for the BQN in other studies [9,11,12].

When using outcome measures such as questionnaires, it is important that the instrument is appropriate for the patient population and setting in which it is used. Although content validity was not specifically evaluated in this study, as it was already established in the original English version [9], it would have been optimal to also repeat this step for the German version of the questionnaire as recommended by Terwee et al. [14]. However, the percentage of the maximum questionnaire score reported by patients at baseline, internal consistency and standardized response means (SRM) are three measures that may provide an indication of the instrument's suitability for use with the patient population under investigation. A comparison of the 3 questionnaire's mean total scores at baseline indicated that 47% of the maximum total score of the BQN was reported by neck pain patients compared to 35% for the NPAD and 28% for the NDI. These results suggest that the BQN is well positioned in the mid range to be able to monitor chiropractic patients' change during treatment either positively or negatively. On the other hand the NPAD and NDI mean scores were in the lower range and may predispose them to floor effects (i.e. baseline scores too low) and potentially underestimate patients' improvement. A further analysis of the mean scores for each of the 10 NDI questionnaire items at baseline identified low mean scores ranging from 0.73 (SD 0.88) to 1.67 (SD 1.22). The exception was pain intensity with a mean score of 2.23 (SD 1.1). This again raises the question of a floor effect and underestimation of patient improvement for the NDI and NPAD.

Another possible interpretation of the previous results is that the NDI is more suited to an acute patient population. Patients are asked to fill out the NDI according to how they feel 'right now' as opposed to the BQN which asks them 'over the past week'. Consequently the NDI may be more suitable for patients whose complaint started within the past few days and the BQN for patients whose pain complaint began possibly a minimum of 5 to 7 days previously. On review, the NPAD would seem to fit in between the NDI and BQN as patients are not given clear instructions (with the exception of pain intensity) as to what time frame to use in order to answer the questions.

Similar to previous studies, the internal consistency of the BQN indicated that all of the 7 questionnaire items were acceptable and well above the 0.2 Cronbach α cut-off point achieving a 0.79 for total pre-treatment scores and 0.82 for total post-treatment scores [9,12,13]. These findings confirm that all of the questionnaire items are relevant to the patient population studied and that they all are necessary, measure the same construct, and contribute to the total score. Nevertheless our results did suggest that question item 7 for pain locus of control, while still important, contributed the least to the BQN total score. This result was also found by Bolton and Humphreys [9] where question 7, although well above the 0.2 Cronbach α, was considerably lower than the other items at pre-treatment and retest. However this was not the case for the Danish translation and validation study for the low back version of the BQ [13]. Further work might be indicated in this area as the question 7 subscale was also difficult to match with the NPAD and impossible to match with the NDI. The correlation between question 7 on the BQN and question 20 on the NPAD prior to treatment, although statistically significant, was much lower (r = .24) than the correlations for all of the other subscales.

The standardized response means (SRM) identified that the BQN is more sensitive to change in this patient population compared to the NDI and NPAD. This corresponds to similar results by Bolton and Humphreys [9] who compared the BQN to the NDI and Copenhagen Neck Functional Disability Scale and Hartvigsen et al. [13] who compared the BQN to the SF-36, although this was done for low back rather than neck pain. Taken together, these results confirm that the BQN is able to detect small clinical changes that are important to neck pain patients, thus emphasizing its utility as a useful and appropriate instrument for assessing this patient population. It has been suggested previously [9] that the BQN is more sensitive to change due to its multidimensional composition. A comparison of the subscales for the 3 questionnaires (Figure 2 and table 5) demonstrates that the NDI does not contain items to assess the cognitive or affective domains, particularly related to psychological impairment (attitudes, beliefs and behaviors) manifested in patients as anxiety, depression, emotions or work related fear-avoidance.

When comparing the SRMs in terms of the sensitivity for each subscale for the 3 questionnaires, it is interesting to note that all seven of the scales (questions) for the BQN are more sensitive than the NPAD or NDI. One possible reason is that the NDI asks patients to respond to each item as they are at present. As patients' pain experience is known to fluctuate, patients' pain experience today may not be representative of their overall neck pain experience [18]. The BQN however asks patients to respond in terms of their average experience over the past week which may be more representative. As mentioned previously, the NDI may be more suited to an acute neck pain population whose pain complaint began in the past few days. The NPAD on the other hand seems to be suitable for patients in between the NDI (current) and the BQN (past week). A possible explanation for this is that the NPAD does not clearly state (other than for pain intensity) what time frame patients should use to answer each of the items. For pain intensity, the instructions are specific, asking for current or worst pain or best pain. However for the other items, it is not clear whether the patient should respond as of now, today, on average over the past week or taking their current episode into consideration.

## Limitations to the study

As mentioned previously, no specific evaluation of content validity of the German version of the BQN was done since it had been established in the English version. Ideally this should have been included in this study in spite of the fact that previous papers reporting on the translation and validation of the BQ into other languages had not included this step [11,13]. Current methodology emphasizes the importance of additional content validity evaluation in the new language [14]. Another limitation of this study is the fact that all testing was done on neck pain patients presenting for chiropractic evaluation and treatment. Whether or not the German version of the BQN is also useful for other neck pain patients should be tested.

## Conclusions

This study confirms that the BQN is a valid, reliable and responsive questionnaire for use in chiropractic patients presenting with neck pain in the German language. Its advantages are that it is short (only 7 questions), more responsive to change and therefore easy to use in the practice or research setting. The results of this study reaffirm that the NDI and NPAD are suitable outcome measures for use in neck pain patients.

## Competing interests

The authors declare that they have no competing interests.

## Authors' contributions

MS: Data acquisition, drafting and revising the manuscript, interpretation of data. CP: Concept and design of the study, analysis and interpretation of data, drafting of results section, revising manuscript. BKH: Concept and design of the study, drafting manuscript discussion section, revising manuscript. All authors read and approved the final version of the manuscript.

## Supplementary Material

Additional file 1 **Appendix**. Final German version of the BQN for validation testing. Click here for file
