# Supplementary material for: Translation and validation of the German version of the Bournemouth Questionnaire for Neck Pain
Source: Chiropr Man Therap. 2012 Jan 25;20:2. doi: 10.1186/2045-709X-20-2 (PMC3398331; doi:10.1186/2045-709X-20-2)
Supplement: Additional file 1 — Appendix. Final German version of the BQN for validation testing. [file 2045-709X-20-2-S1.PDF]

Bournemouth Fragebogen (7 Fragen)

Die folgenden Fragen wurden zusammengestellt, um mehr über Ihre Nackenschmerzen und deren Auswirkungen herauszufinden. Bitte beantworten Sie ALLE Fragen, indem Sie auf der jeweiligen Skala diejenige Zahl mit einem Kreis markieren, welche am besten beschreibt, wie Sie sich fühlen:

1. Wie stark würden Sie Ihre Nackenschmerzen der **letzten Woche** im Durchschnitt einstufen?

0 1 2 3 4 5 6 7 8 9 10
Keine Schmerzen — Schlimmst mögliche Schmerzen

2. Wie stark wurde Ihr Tagesablauf **letzte Woche** von Ihren Nackenschmerzen beeinträchtigt? (Hausarbeit, Waschen, sich ankleiden, etwas aufheben, lesen, Auto fahren)

0 1 2 3 4 5 6 7 8 9 10
Gar nicht beeinträchtigt — Konnte gar nichts erledingen

3. In welchem Ausmass haben Ihre Nackenschmerzen Sie während der **vergangenen Woche** daran gehindert, an Freizeit-/Familienaktivitäten oder anderen sozialen Aktivitäten teilzunehmen?

0 1 2 3 4 5 6 7 8 9 10
Teilnahme uneingeschränkt möglich — Teilnahme nicht möglich

4. Wie unwohl/unruhig (verspannt; nervös; reizbar; Mühe, sich zu konzentrieren und zu entspannen) haben Sie sich während der **vergangenen Woche** gefühlt?

0 1 2 3 4 5 6 7 8 9 10
Gar nicht — Extrem unwohl/unruhig

5. Wie deprimiert (niedergeschlagen, traurig, pessimistisch, unglücklich) haben Sie sich während der **vergangenen Woche** gefühlt?

0 1 2 3 4 5 6 7 8 9 10
Überhaupt nicht deprimiert — Extrem deprimiert

6. Wie stark wurden Ihre Nackenschmerzen durch Ihre Arbeit (zu Hause und am Arbeitsplatz) in der **letzten Woche** beeinflusst?

0 1 2 3 4 5 6 7 8 9 10
Hat die Nackenschmerzen nicht verschlimmert — Die Schmerzen wurden sehr viel schlimmer

7. In welchem Ausmass konnten Sie selber Ihre Nackenschmerzen in der **vergangenen Woche** unter Kontrolle halten/vermindern?

0 1 2 3 4 5 6 7 8 9 10
Vollständige Kontrolle möglich — Keine Kontrolle allein möglich
